# Identifying risk factors for household burdens of road traffic fatalities: regression results from a cross-sectional survey in Taiwan

**DOI:** 10.1186/s12889-016-3813-3

**Published:** 2016-11-29

**Authors:** Lanying Huang

**Affiliations:** Graduate School of Criminology, National Taipei University, 151 University Rd., San Shia District, New Taipei City, 23741 Taiwan

## Abstract

**Background:**

Road traffic fatalities (RTF) are among the top ten causes of deaths in the world. The risk factors for RTF fatal victims have been extensively characterized, but studies of household burden of RTF have been very few in number. Accordingly, this article investigates post-crash impacts on RTF victims’ family members, including the adverse impacts of lost income, occupational disruption, unfavorable family dynamics, and residential relocation.

**Methods:**

Survey data from 1291 RTF family members interviewed in Taiwan in 2012 provide the evidence of impact used in this article. Twelve variables related to the family member’s socio-demographic background were used to predict the scope of the adverse impact of a fatal crash in regression models developed for this analysis.

**Results:**

RTF victims’ spouses with relatively low personal incomes and strong dependence upon the crash victims were found to be most likely to experience a marked decrease in post- crash quality of life. RTF victims’ family members who lived with few other adult cohabitants and had more juvenile dependents and were emotionally dependent on the victims were found to be quite likely to experience post- crash setbacks in occupational stability. RTF victims’ family members who were emotionally dependent on the victims were found to be more likely to experience major family life disruptions. The younger the RTF victims’ family members, and the more years since the crash, the higher the likelihood of residential relocation taking place.

**Conclusions:**

The results noted help identify those RTF victims’ families that will most likely be adversely affected by the crash. The true societal costs of RTF crashes should include the adversities suffered by the fatal crash victims’ families. Social welfare policies, mental health support, and timely supplemental resources should be made available to those surviving families most at risk of major life disruptions.

## Background

### Heavy cost of road traffic crashes in low- and middle-income countries

As the eighth leading cause of deaths globally, road-traffic fatalities (RTF) took more than one million lives each year over the course of the last decade [1]. The estimated costs to society of traffic crashes are believed to be substantial, ranging from 1 to 3% of GDP [2]. The World Health Organization’s most recent report on the matter predicts that RTFs will become the fifth leading cause of death by 2030 if nations do not hasten to take more progressive actions to address the problem than they have to date.

The ratio of fatal to non-fatal injury road-traffic crashes was estimated to be around 1:20 [3]. Large disparities in this ratio exist across nations with low- and middle-income countries exhibiting more than twice the fatality rate of high income countries, and the gap is expected to increase as passenger vehicles in the prosperous countries become markedly better equipped with safety features [4]. From a global perspective, it is clear that greater effort must be devoted to reducing traffic fatalities in low- and middle-income countries [5, 6].

Unfortunately, those countries which are experiencing the most rapid rates of increase in RTFs tend to not devote much attention to road safety enhancement, and governmental resources needed to address the problem globally such as engineering improvements, more effective traffic law enforcement and more efficacious public education are not evenly distributed among nations [2].

### Substantial research of consequences of road traffic crashes on victims but not on victim's families

Our knowledge of both the immediate and the long-term physical and psychological harm attributable to road traffic crashes to the victims is fairly extensive [7–17]. Previous empirical studies have confirmed that road traffic crash victims often suffer a combination of physical, psychological, financial, social, and legal adverse impacts from their crashes. Although physical lesions and broken bones often are healed with the passage of time, psycho-social complications arising from road traffic crashes often persist for a rather long time. Mayou and Bryant [7] reported that a third of victims of injury vehicle crashes were still suffering psychological, social and legal adversities one year after their crashes. In a Swedish study, over half of road traffic accident victims reported at least one psychological symptom after a 2-year follow-up [8].

Past studies have also investigated a number of risk factors associated with adverse psycho-social impacts arising from road traffic crashes for their victims, finding variations in impacts across various socio-demographic traits of the victims. For example, a survey of victims conducted in Sweden showed that females tend to report more adverse consequences from their road traffic crashes than men [9]. Another 6-month follow-up study done in France found that the risk of post-traumatic stress disorder (PTSD) was higher among female victims than male victims [10]. The 2-year follow-up survey carried out in Sweden noted above found that females over 40-years in age were more likely to develop psycho-social complications than were younger women involved in comparable crashes [8]. Age for both men and women was also found to be a strong predictor of serious injury in several studies [18, 19]; however, age was not associated with the likelihood of developing chronic PTSD [10, 11]. Some studies went beyond immediate individual health or psychological impacts to the consideration of long-term economic consequences suffered by road traffic crash victims and their families.

The majority of literature of the impact and consequences of road-traffic crashes concentrates on studying the surviving injured victims of serious collisions [8, 12–16]. While the impact of fatal crashes on the victim’s family members has seldom been the focus of systematic investigation, family members of persons killed in traffic crashes might well be imagined to be greatly affected in many ways, both directly and indirectly. For example, in one published research study of a severely injured crash victim, the family of the seriously injured driver, and the family of victims who died in the crash (RTF victims) were all found to be rather seriously *traumatized* as a group, without great difference across these distinct groups of people associated with the RTF event [17]. In Mohan’ s words, the adverse outcomes of serious injury or death of a family member can be ‘permanent and soul destroying for individuals and possibly for the larger community’ [5]. Mohan therefore calls for more research work to understand this issue. Because of this lack of needed research, this article explores the after-crash impact upon the families of RTF victims in Taiwan in order to help fill this gap in our collective knowledge.

### High density of motorcyclists and the Crime Victim Protection Act in Taiwan

The annual road traffic fatalities per 100,000 inhabitants of Taiwan, a high-income country, was 12.4 in 2014, a figure which is somewhat lower than the world average of 17.5, but which is much higher than in the neighboring country of Japan, whose rate is only 4.7 per 100,000. The higher rate relative to Japan stems in some part from the difference in the number of motorcycles per square kilometer; the figure for Taiwan was 392.2 in 2013, a number which is more than 10 times that of Japan [20]. Motorcyclists are among the most vulnerable road users according to the Global Status Report on Road Safety [1]. Among all the drivers who lost their lives within 24 hours of an accident in Taiwan, 78.1% are motorcycle drivers; the corresponding figure for Japan in 17%. Male victims constitute 70.8% of all fatalities of traffic road crashes [20]. Many of those RTF victims were the heads of male-headed households and the primary "breadwinners" in those households.

In Taiwan, vehicle crashes resulting in a fatality are considered criminal acts, and the victims are considered victims of crime. The families of RTF victims, along with other dead victims of violent crimes, are fully entitled to crime victim services according to the Crime Victim Protection Act. Local victim services, at this writing staffed by 54 full-time workers and 823 volunteers, are available in 21 district prosecution offices spread across Taiwan. Service provided include securing and forwarding information and assistance according to each victim's needs during different time phases. Services such as shelter provision, legal aid, social services, medical aid, occupational therapy, physical rehabilitation, emergency compensation and even longer-term supplemental support, and when possible restitution are all involved. In order to develop effective evidence-based post-fatality interventions for victims' family members who need support and rehabilitation, an understanding of RTF surviving family members' ordeals from the population of people who were impacted by a traffic fatality and reported on their own real life victimization experience to the crime victim service offices is helpful.

### Studying effects of RTF on victims' households using data from the crime victim services in Taiwan

The adverse impacts of RTFs on victims and associated parties are measured by reference to both objective and subjective criteria [21]. Objective measures are mainly those of material loss such as the costs of vehicle replacement, medical care, higher insurance premiums, and consequently the reduction of personal and/or household income [16]. Subjective measures concern principally the psychological effects of crash involvement on quality of life; fear, stress-related anxieties, and sleep interruption from crash re-enactment dreams and PTSD in some cases are the types of psychological outcomes typically documented. Although some previous studies have employed both subjective and objective measures of outcomes, research studies usually emphasize the more easily quantified objective measures, namely the monetary calculation [9, 12, 16].

As I pointed out that, compared to the RTF victims, the adversities suffered by the families of RTF victims have not been well addressed in the research literature. Systematic research in this area can help lessen the burdens of RTF accidents by prioritizing societal response to the parties most at risk of adverse consequences from the loss of a family member. Toward this goal, the present study aims to identify the impacts of a RTF crash on victims' households, and to document the risk factors that incur household burdens using cross-sectional survey data collected in Taiwan. Household burdens are here defined as victimization impacts on family members of RTFs which have adverse effects on family functions. We hypothesize that the impacts in question fall into four distinct types: family relations, occupational life, family income, and residentail relocation. Potential risk factors that might be used to predict the scope and severity of the adverse impacts includes: family members' demographic characteristics, their social-economic position, their familial situation, the time gone by since the crash, and their emotional attachment to the fatality victim.

## Methods

### Data

Data used in this article come from a cross-sectional survey of victims and victims’ families in Taiwan conducted under the auspices of a government (Department of Prevention, Rehabilitation and Protection, Ministry of Justice)-contracted research project carried out by a market research and consultation firm. The Case Management System of the Association for Victims Support, which is a Taiwan government (Ministry of Justice)-funded national institution responsible for victim services, holds information of all the victims who have registered in local victim support offices since 2001. The number of RTF victims’ families with full contact information in the system was 4,561. They were contacted by trained interviewers, and 1291 (28.3%) of them consented to a face-to-face interview and completed a structured, printed questionnaire during the period between July and November of 2011.

Personal background information in the survey questionnaire includes age, gender, formal education, occupation, job position and personal income. Formal education is assessed in terms of five levels, ranging from 'elementary or below' to 'graduate school or above'. The occupation question features 21 categories reflecting the major sectors of the Taiwan economy, ranging from farming to entertainment and including the category of unemployed. ‘Others’ is among the 21 response options if none of the listed answers fits the interviewee’s occupation. Note that the answer options include ‘no idea’ and ‘no answer’ as the labeled options. Position refers to an interviewee’s job title in his/her occupation, and this item has 10 categories as shown in Table 1. The personal income item requests the interviewee’s monthly income in NT dollars. A category is to be selected from the deciles between 0 and 100 K.

**Table 1:** Socio-demographic information of the RTF victims’ family members

Factor/levels	Number	Percent
Gender		
male	731	56.6
female	560	43.4
Education		
elementary or below	161	12.5
junior high	302	23.4
senior high	494	38.3
college	292	22.6
graduate or above	42	3.3
Occupation		
farming	72	5.6
mining	1	0.1
manufacturing	204	15.8
power	8	0.6
water	5	0.4
construction	78	6
retailing	109	8.4
transportation	37	2.9
restaurant	66	5.1
information	33	2.6
finance	44	3.4
real estate	7	0.5
technology	49	3.8
supporting	27	2.1
public services	49	3.8
education	30	2.3
healthcare	39	3
entertainment	26	2
others	64	5
no idea	3	0.2
no answer	4	0.3
unemployed	336	26
Position		
manager	81	6.3
professional	106	8.2
technician	78	6
supporting	57	4.4
sales	220	17
craftsman	42	3.3
mechanical	37	2.9
labor	331	25.6
no answer	3	0.2
unemployed	336	26
Personal income		
[0, 10 K)	54	4.2
[10 K, 20 K)	186	14.4
[20 K, 30 K)	300	23.2
[30 K, 40 K)	158	12.2
[40 K, 50 K)	84	6.5
[50 K, 60 K)	54	4.2
[60 K, 70 K)	15	1.2
[70 K, 80 K)	11	0.9
[80 K, 90 K)	5	0.4
[90 K, 100 K)	17	1.3
no idea/no answer	71	5.5
unemployed	336	26

Information about the interviewee's family was also queried in the survey. This section of the survey includes information on cohabitants and dependents present in the household. In the case of cohabitants, the possible answers are: 1) parents, including father and/or mother; 2) spouse, including partner; 3) unmarried offspring; 4) married offspring and their spouses; 5) grandchildren; 6) friends; 7) other relatives; 8) living alone; and 9) refuse to answer. Multiple answers could be chosen on this item. In the case of dependents, interviewees answered how many dependents younger than 18 he/she was living with at the time of the fatal crash.

The survey also included an item on the interviewee's relation with the victim of the fatal crash. In the case of family relationship, answers include parents, spouse, offspring, grandchild, sibling and others. In the area of emotional ties to the victim, interviewees were asked to pick one among the five levels of emotional dependence he/she had on the victim. Similarly, in the area of financial ties, one of the five options ranging from very much financially dependent to not dependent at all was to be chosen.

Finally, two questions regarding average household income were posed. One survey item concerns current average monthly household income, and the other item asked about the average monthly household income at the time of fatal traffic crash. Another question asked about impact of the RTF crash on the family member's occupational activities. Possible answers in the questionnaire included: 1) psychological pressure from colleagues/supervisors; 2) unable to work as usual because of lawsuit or funeral; 3) interpersonal relations affected; 4) forced to quit job by the employer; 5) was discriminated against when returning to job market; 6) unable to work normally because of emotional turmoil; 7) chose to quit in order to take care of family members; 8) others; 9) no impact at all; and 10) no idea or refuse to answer. Another questionnaire item asked about the impact of the crash on family life, with answers including: 1) unable to sustain basic living because of affected family income; 2) safety was threatened; 3)family members feel gloomy, losing peace of mind; 4) family members blame one another; 5) chilled family relationship; 6) afraid of being asked about the event, hence affecting social life; 7) difficulty taking care of children; 8) others; 9) not affected at all; 10) no idea or refuse to answer. Finally, the interviewee was asked whether he or she had relocated because of the crash.

### Statistical analysis

The data collected in the survey enable us to test the hypotheses on the four types of RTF impacts and to identify the risk factors associated with the impacts on the victim's household through the use of logistic regression: impact on 1) household income; 2) occupational activities; 3) family life; and 4) residential relocation. Specifically, a decrease in the average household incomes between the time of crash and the time of interview was categorized as an impact on the interviewee's household income. Otherwise, there was no impact. Note that income was not adjusted for inflation because the inflation rate has been low in Taiwan in the past 20 years. Similarly, choice of no affect to the questions about occupational activities, family life and residential relocation indicates no impact. Other chosen answers indicate an impact. The dichotomized impact responses enable the calculation of impact probability, p, from the survey interviewees. Next, changes in each of the four types of household burdens are modeled by the use of 12 independent variables representing potential risk factors in the following way:

$$ \mathrm{Log}\left(p/\left(1\hbox{--} p\right)\right)=\mathrm{gender}+\mathrm{age}+\mathrm{relation}+\mathrm{education}+\mathrm{occupation}+\mathrm{position}+\mathrm{personal}\ \mathrm{income}+\mathrm{cohabitants}+\mathrm{dependents}+\mathrm{years}\ \mathrm{past}+\mathrm{emotional}\ \mathrm{ties}+\mathrm{financial}\ \mathrm{ties} $$

In the regression model, age, personal income, number of cohabitants, number of dependents, years since RTF crash, emotional ties and financial ties were treated as numerical variables; the rest were treated as categorical. Multi-collinearity among the 12 explanatory variables was assessed using Pearson correlation matrix, p, and the variance inflation fator (VIF). Education levels were found negatively correlated with position (*p* = -0.44) and age (*p* = - 0.32), and positively correlated with household income (*p* = 0.33); position was negatively correlated with household income (*p* = -0.31); financial ties were positively correlated with emotional ties (*p* = 0.33) but negatively correlated with gender being male (*p* = -0.35). The rest of the pair-wise correlations were less than 0.3 in magnitude. Despite the weak correlations noted, the generalized variance inflation factors, GVIF^1/(2df)^ for the 12 explanatory variables were all less than 1.3, permitting the use of the 12 variables as independent variables in the logistic regression model [22, 23].

## Results

The ages of the interviewees range from 20 to 85 years, with an average of 47.5 years and standard deviation of 10.1 years. A display of the descriptive statistics for the personal background information is set forth in Table 1, which indicates that the typical victim's family member who was interviewed was a male, with a senior high school degree, working as a laborer in the manufacturing sector with a monthly wage between 20 and 30 thousand NT dollars.

The descriptive statistics set forth in Table 2 indicate that the majority of the responding victims’ family members lived with one or two generations of cohabitants, with approximately two juvenile dependents.

**Table 2:** Family-related information of the RTF victims’ family members

Factor/levels	Number	Percent
Multiple cohabitants		
0	84	6.5
1	476	36.9
2	558	43.2
3	152	11.8
4	20	1.5
6	1	0.1
Number of dependents (persons)		
0	633	49
1	290	22.5
2	289	22.4
3	61	4.7
4	13	1
5	1	0.1
6	2	0.2
7	1	0.1
NA	1	0.1

Findings reported in Table 3 indicate that most interviewees were either the child, spouse, or parent of the RTF victims, and that they were most often both emotionally and financially heavily dependent on the victims.

**Table 3:** Relation information of the RTF victims’ family members

Factor/levels	Number	Percent
Relation		
parent	372	28.8
spouse	384	29.7
child	404	31.3
grandparent	1	0.1
grandchild	6	0.5
sibling	87	6.7
others	37	2.9
Emotional ties		
very much	710	55
very	390	30.2
moderately	91	7
not very	34	2.6
not	61	4.7
NA	5	0.4
Financial ties		
very much	334	25.9
very	224	17.4
moderately	79	6.1
not very	72	5.6
not	581	45
NA	1	0.1

**Fig. 1:**
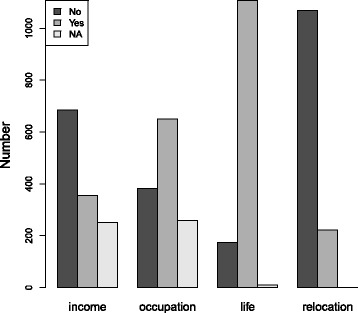
RTF accident adverse impacts on victims’ family members: Number reporting adverse effects on household income, occupational activities, family life, and residential relocation

The proportions of RTF victim family members who reported adverse outcomes with respect to household income, occupational activities, family life and residential relocation are 27.6, 50.4, 85.9 and 17.2%, respectively; those results are set forth in Fig. 1. It is noteworthy that the lingering adverse effects of the RTF crash most often cited was that of impact on family life.

**Fig. 2:**
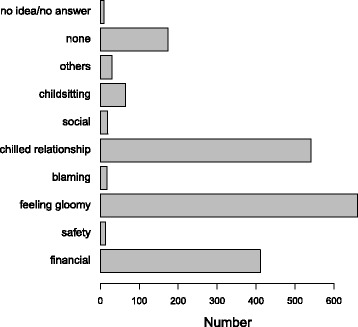
RTF accident adverse impacts on family life: Number reporting stress associated with chilled relationships, gloomed feeling and financial woes

**Fig. 3:**
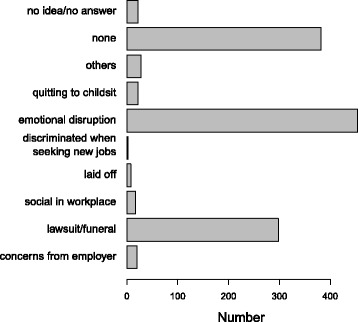
RTF accident adverse impacts on occupational activities: Number reporting stress associated with emotional turmoil and lawsuit/funeral affairs

Figure 2 displays the detailed pattern of responses on survey items dealing with post-RTF family dynamics. In the area of occupational activities effects, emotional turmoil was reported to be the major negative impact on one’s working life, as shown in Fig. 3.

Table 4 displays the results of the logistic regression with respect to the four types of impacts. The most noteworthy findings are as follows. The odds of reduced household income for victim’s spouse are 75.5% greater than the odds for victim’s parents; the odds of reduced household income decrease by 16.3% per each 10 K increase in the survey respondent’s personal income; and the odds decrease by 30.8% per level decrease in the interviewee’s financial ties to the victim. With respect to the occupational activities impact, the odds decrease by 22.8% with each increase in the number of household adult cohabitants; the odds increase, however, by 21.2% for each increase in the number of juvenile dependents in the household; and the odds decrease by 19.9% per level decrease in the strength of emotional ties to the victim. In the area of family life impact, the odds are 65.1% lower for restaurant workers than the odds for farming workers; and the odds decrease by 29.5% per level decrease in the strength of emotional ties to the victim. Finally, in the area of the residential relocation impact, the odds decrease by 2.7% per year increase in the interviewee’s age; the odds for mechanical workers are 89.8% lower than the odds for managers; and the odds increase by 8.9% per year since the RTF crash.

**Table 4:** Results of logistic regression prediction of RTF impact on victim’ family

	Reduced Family income				Occupation Impact				Family Life Impact				Relocation			
	OR^α^	95% CI^β^	*p* value^γ^	sig^δ^.	OR	95% CI	*p* value	sig.	OR	95% CI	*p* value	sig.	OR	95% CI	*p* value	sig.
Gender																
male	1.000				1.000				1.000				1.000			
female	1.055	0.672–1.654	0.817		1.090	0.756–1.573	0.644		1.431	0.864–2.369	0.164		1.484	0.933–2.358	0.095	< 0.1
Age	0.996	0.973–1.019	0.710		0.998	0.979–1.017	0.796		0.992	0.967–1.017	0.524		0.973	0.951–0.996	0.021	< 0.05
Relationship																
parent	1.000				1.000				1.000				1.000			
spouse	1.755	1.030–2.992	0.039	<0.05	0.865	0.540–1.385	0.546		1.750	0.851–3.600	0.128		1.693	0.947–3.027	0.076	< 0.1
offspring	0.752	0.438–1.290	0.300		0.945	0.645–1.384	0.772		0.834	0.511–1.362	0.468		0.764	0.445–1.313	0.330	
grandparent	0.000	0-Inf	0.993		25677839.540	0-Inf	0.994		6823240.234	0-Inf	0.995		0.000	0-Inf	0.997	
grandchild	0.000	0-Inf	0.984		0.820	0.129–5.229	0.834		4934945.060	0-Inf	0.988		1.431	0.137–14.937	0.765	
sibling	1.107	0.495–2.474	0.805		0.939	0.505–1.744	0.841		0.627	0.300–1.311	0.215		0.692	0.292–1.640	0.403	
others	0.981	0.275–3.503	0.977		0.700	0.269–1.819	0.464		0.733	0.248–2.170	0.575		0.672	0.180–2.517	0.556	
Education																
elementary or under	1.000				1.000				1.000				1.000			
junior high	1.126	0.546–2.319	0.748		1.275	0.707–2.300	0.419		1.874	0.865–4.062	0.111		1.251	0.573–2.732	0.574	
senior high	1.245	0.613–2.529	0.544		1.157	0.649–2.062	0.621		1.758	0.821–3.764	0.146		0.981	0.455–2.114	0.960	
college	1.584	0.698–3.590	0.271		1.045	0.538–2.033	0.896		1.236	0.517–2.957	0.634		1.452	0.620–3.400	0.391	
graduate or above	2.387	0.539–10.569	0.252		0.860	0.313–2.364	0.771		1.028	0.282–3.752	0.967		1.062	0.274–4.118	0.930	
Occupation																
farming	1.000				1.000				1.000				1.000			
mining	0.000	0-Inf	0.993		11293292.180	0-Inf	0.995		3487607.413	0-Inf	0.995		0.000	0-Inf	0.997	
manufacturing	1.071	0.457–2.512	0.875		0.786	0.423–1.462	0.447		0.669	0.287–1.555	0.350		1.566	0.598–4.101	0.361	
power	0.385	0.036–4.136	0.431		1.109	0.231–5.336	0.897		2591290.295	0-Inf	0.985		0.000	0-Inf	0.991	
water	0.000	0-Inf	0.983		10249867.050		0.988		3025100.794	0-Inf	0.988		0.000	0-Inf	0.993	
construction	0.784	0.288–2.139	0.635		1.999	0.964–4.144	0.063	< 0.1	0.756	0.304–1.881	0.548		1.382	0.456–4.183	0.567	
retailing	0.584	0.216–1.576	0.288		1.343	0.631–2.858	0.444		0.451	0.166–1.224	0.118		1.177	0.400–3.468	0.767	
transportation	0.530	0.147–1.912	0.332		1.381	0.562–3.395	0.482		0.877	0.264–2.909	0.830		1.239	0.300–5.119	0.768	
restaurant	1.008	0.358–2.836	0.988		1.600	0.692–3.696	0.272		0.349	0.124–0.987	0.047	< 0.05	1.183	0.380–3.677	0.772	
information	0.582	0.158–2.149	0.417		1.538	0.565–4.184	0.399		0.630	0.171–2.326	0.488		0.976	0.246–3.872	0.973	
finance	1.018	0.307–3.370	0.977		1.570	0.596–4.140	0.361		2.092	0.393–11.151	0.387		1.014	0.280–3.674	0.983	
real estate	1.824	0.262–12.686	0.543		6421323.261	0-Inf	0.986		0.439	0.042–4.537	0.489		2.664	0.376–18.880	0.327	
technology	0.886	0.274–2.867	0.840		0.876	0.383–2.004	0.754		1.012	0.322–3.181	0.984		1.368	0.402–4.657	0.616	
supporting	0.674	0.185–2.457	0.550		0.372	0.138–1.004	0.051	< 0.1	0.410	0.113–1.484	0.174		0.000	0-Inf	0.983	
public services	0.589	0.162–2.148	0.423		0.541	0.227–1.292	0.167		0.720	0.229–2.260	0.573		1.180	0.335–4.163	0.796	
education	1.106	0.306–3.998	0.877		0.443	0.157–1.248	0.124		1.256	0.211–7.463	0.802		1.368	0.350–5.347	0.652	
healthcare	0.610	0.184–2.022	0.419		1.072	0.429–2.677	0.882		1.237	0.323–4.741	0.756		3.164	0.989–10.125	0.052	< 0.1
entertainment	1.155	0.319–4.181	0.827		0.909	0.323–2.559	0.857		0.557	0.138–2.250	0.412		2.162	0.603–7.744	0.236	
others	1.032	0.388–2.740	0.950		0.859	0.399–1.850	0.698		1.846	0.512–6.663	0.349		1.392	0.460–4.213	0.558	
no idea	0.000	0-Inf	0.992		0.000	0-Inf	0.991		0.000	0-Inf	0.989		0.000	0-Inf	0.995	
no answer	NA	NA	NA	NA	7212970.713	0-Inf	0.995		1067322.415	0-Inf	0.995		0.000	0-Inf	0.997	
Job Position																
manager	1.000				1.000				1.000				1.000			
professional	0.975	0.371–2.564	0.960		1.307	0.656–2.603	0.446		1.455	0.571–3.709	0.433		0.722	0.305–1.713	0.461	
technician	1.046	0.390–2.804	0.929		1.299	0.623–2.708	0.485		0.844	0.330–2.159	0.723		0.816	0.334–1.994	0.656	
supporting	1.260	0.439–3.617	0.668		0.686	0.321–1.467	0.331		1.088	0.369–3.206	0.879		0.556	0.207–1.490	0.243	
sales	1.332	0.581–3.055	0.499		1.750	0.967–3.168	0.065	< 0.1	2.206	0.985–4.943	0.055	< 0.1	0.771	0.373–1.596	0.484	
craftsman	0.791	0.234–2.677	0.707		0.738	0.317–1.721	0.482		0.456	0.161–1.285	0.137		1.006	0.338–2.996	0.992	
mechanical	0.823	0.248–2.730	0.750		0.938	0.390–2.254	0.886		0.815	0.249–2.667	0.735		0.102	0.012–0.877	0.038	< 0.05
labor	0.975	0.425–2.238	0.953		0.884	0.496–1.576	0.677		0.904	0.420–1.946	0.796		0.576	0.272–1.220	0.150	
no answer	NA	NA	NA	NA	0.000	0-Inf	0.992		0.000	0-Inf	0.995		0.433	0-Inf	1.000	
Personal income	0.837	0.719–0.974	0.022	< 0.05	0.971	0.910–1.036	0.379		1.019	0.935–1.112	0.664		0.969	0.884–1.062	0.501	
Cohabitants	0.873	0.665–1.147	0.330		0.772	0.627–0.950	0.015	< 0.05	0.964	0.729–1.276	0.799		0.999	0.765–1.305	0.994	
Dependents	1.067	0.883–1.290	0.499		1.212	1.027–1.431	0.023	< 0.05	1.019	0.816–1.273	0.866		0.847	0.685–1.046	0.123	
Years since RTF	1.026	0.958–1.099	0.465		1.025	0.966–1.089	0.413		0.973	0.896–1.057	0.522		1.089	1.017–1.165	0.014	< 0.05
Emotional ties	0.976	0.782–1.218	0.829		0.801	0.686–0.935	0.005	< 0.01	0.705	0.587–0.846	0.000	< 0.001	1.008	0.819–1.241	0.939	
Financial ties	0.692	0.607–0.789	0.000	< 0.001	0.929	0.833–1.037	0.189		0.868	0.744–1.013	0.072	< 0.1	0.965	0.838–1.111	0.618	

α. OR = Odds ratio

β. 95% CI = 95% confidence interval

γ. *p* value = logistic regression *p*-value

δ. sig = statistical significance

## Discussion

This paper used survey data from 1291 family members of RTF victims to present the risk factors associated with four types of potential adverse impacts. Only 27.6% of the family members reported reduced household income post-crash. If the family member was a spouse or had close financial ties with the victim, the possibility of income loss was significantly higher. However, the family member who had a high personal income was significantly less likely to report reduced family income.

Unlike the previous study of injured victims reported by Tournier et al. [12], this study did not find evidence of a temporal effect for the reporting of income loss in the family whereby the loss diminished over time post-crash. In the case of RTF family members the financial loss appears to be persistent and long-lasting. This is probably the case because the RTF represents a permanent loss of productivity in the family which cannot be remedied by occupational therapy and re-employment post-crash. In addition, in the case of injured victims, the medical costs associated with injury recovery likely decrease over time in most cases, easing the financial difficulties over time.

About half (50.4%) of the family members surveyed underwent post-crash occupational turmoil, mostly due to emotional disruptions or lawsuit/funeral affairs. Emotional ties with the victim significantly increased the risk of occupation impact. More children present in the household was also associated with an adverse occupational activities impact. Living with relatives and/or friends, nevertheless, reduced the risk of adverse occupational activities impact.

Tournier et al. [12] found that intention to lodge a complaint was a risk factor for adverse occupational impacts. This implies that placing complaints, or filing lawsuits, might wear upon the injured person and their family, and make work life circumstances more difficult. Having more children in the household might be a risk factor for occupational impact because of the increased childcare burdens arising after the RTF crash. It is interesting to note that previous research has found that women with children were more likely than childless women to develop psychological complications after a serious injury crash [8]. This finding suggests that having responsibilities for raising children is likely a risk factor for both psychological complications and occupational life management, with the former being predictive of the later. In contrast, multiple adult cohabitants can be considered an indicator of likely social support. People with more generations of cohabitants in the household likely can receive more forms of assistance from other members in the family to compensate for the functions previously provided by the lost member.

In the area of work life and private life connection, a two-year follow-up survey of 134 serious traffic crash injury victims conducted in Göteborg showed that people who were unable to return to their prior jobs post-crash developed complications in their recoveries and suffered major adjustment problems [8]. The implication is that timely assistance is crucial in terms of maintaining a family system which could help victims cope with short term and longer term demands upon RTF victim families.

It was found that over 85% of family members reported at least one kind of family life adverse impact. Among those impacts, *psychological* (i.e. feeling sad), *relational* (i.e. chilled family relationships) and *financia*l travails were most often noted. The logistic regression model results pertaining to family life impact indicate that the strength of emotional ties with the RTF victim predicts the risk of suffering from adverse family life impacts. Interestingly, family members who were in restaurant-related occupations had less of a risk of experiencing adverse family life impacts than others.

The RTF victim family survey findings suggest that adverse familial life impacts did not fade over time, nor were those negative impacts associated with age, gender, or other socio-demographic factors. The key element in this area was that of emotional ties with the RTF victim; the more strongly connected by emotional ties the more likely it is that family members will suffer from adverse family life impacts.

Less than one in five survey respondents reported post-crash residential relocation (17.2%). Three factors were found to have significant effects on relocation, those being age, job position, and years since the RTF crash. Family members who were of older and who were mechanic workers were less likely to move. The more years since the crash, the more likely the family members were to report having relocated.

Relocation is a difficult area to interpret since it might be associated with the familial or occupational impacts of crashes, or it might represent a normal coping strategy to deal with occupational, familial, or financial difficulties generally unrelated to the RTF crash. The meaning and effects of relocation for RTF victims are worth exploring in greater detail than is possible with the survey data available for this study.

## Conclusion

Since the existing literature is mostly concerned with injury risks and the outcomes of post-crash readjustment, our knowledge of the impact of RTF crashes on victims’ families is very limited. Families which have lost members as a consequence of a fatal crash are *hidden victims* who must undergo a lengthy recovery process, and they would likely benefit greatly from an established long term institutional response. This research addresses four noteworthy commonplace post-crash adverse impacts – namely, family dynamics, occupational activity disruptions, financial woes, and social dislocation. It also develops four predictive models for gaining insight into how different hidden victims of RTF crashes come to suffer hardships from those fatal crashes.

In comparison with previous literature centered on injured victims, it is disturbing to find that post-crash occupational disruption among RTF victim families is nearly as prevalent as among seriously injured traffic crash victims. This suggests that the true costs of serious traffic crashes are not merely restricted to individual health recovery of survivors of serious crashes and the replacement of lost equipment. Perhaps the true cost of RTF crashes should include the psycho-social costs and adverse impacts suffered by the victim and the victim’s family. Road traffic crashes carry a very high socio-economic cost for the society and the families affected in part because traffic crash injuries and casualties generally occur in victims’ mid-life years [13, 24].

Properly addressing these high societal costs requires government action of at least two types. First, there must be continued efforts to reduce the incidence of traffic collisions and prevent the mortalities of road traffic crashes through safer roadways, safer cars, more effective law enforcement of traffic safety laws, and better public education of drivers. Second, there is a need to restore the quality of life and repair the emotional damage done to the families suffering from the outcomes associated with RTF crashes. In this regard, helping the families of RTF crashes in a timely way would likely greatly reduce the long term societal costs of fatal crashes.

Taiwan has had appropriate legislation in place for a long time to address the five primary risk factors for road traffic crashes – namely, excessive speed, drinking and driving, use of helmets on motorbikes, use of seatbelts, and use of child safety restraints. Nevertheless, road traffic crash statistics bear witness to the fact that the ongoing enforcement of these laws, even with the addition of closed-circuit TV monitors in key locations, remains far from optimal.

With respect to addressing the societal costs arising from the needs of crash survivors, current rehabilitation programs principally target injured persons and pursue the goal of crash victim reintegration into the workforce and broader society. It is argued here that the families of the fatally injured victims of RTF crashes should also be included in both the estimation of societal costs of injury traffic crashes and after-crash services.

This article provides a glimpse of the baseline service needs of RTF crash families in a high-income country. The question of documenting the quality of life impacts on victims’ families after fatal traffic crashes deserves more research, and more resources are needed to prevent such crashes and provide timely assistance to affected persons after they occur. This article provides a picture of the scale of the problem, and highlights the importance of collecting data from the family members of road traffic crash victims.

This study does a fair job of providing perspective on the problem at hand, but its scope is rather limited. The response rate is low and unavailability of non-respondents' information keeps us from assessing/correcting potential biases. For example, results of the descriptive statistics show that many respondents worked as laborers and it would be interesting to know the occupation of those who did not respond. On the other hand, since the design of the current study compares pre- with post-crash status of the same respondent, it is self controlled and thus immune to bias in population stratification. The survey method constraints the range of questions that can be asked and limits the depth of understanding of responses provided. In some areas, such as the long-term psychological complications associated with PTSD, adverse outcomes were not measured in the survey. The current study differentiated four different types of potential adverse impacts and investigated their correlates in predictive statistical models using logistic regression. However, previous research suggests that victims’ social consequences often interacted with and reinforced psychological and medical impairments [9]. It is hoped that these limitations can be overcome in future research, and that evidence-based ameliorative interventions can be made with RTF victims’ families at high risk of adverse impacts. If such interventions are developed, it is hoped that the outcomes of those interventions are monitored for determining their effectiveness vis-à-vis ongoing cost/benefit analyses.

## Abbreviations

RTF: Road traffic fatalities
